# What is the origin of the ectopic beat?

**DOI:** 10.1007/s12471-015-0656-y

**Published:** 2015-02-12

## Answer

**Fig. 1:**
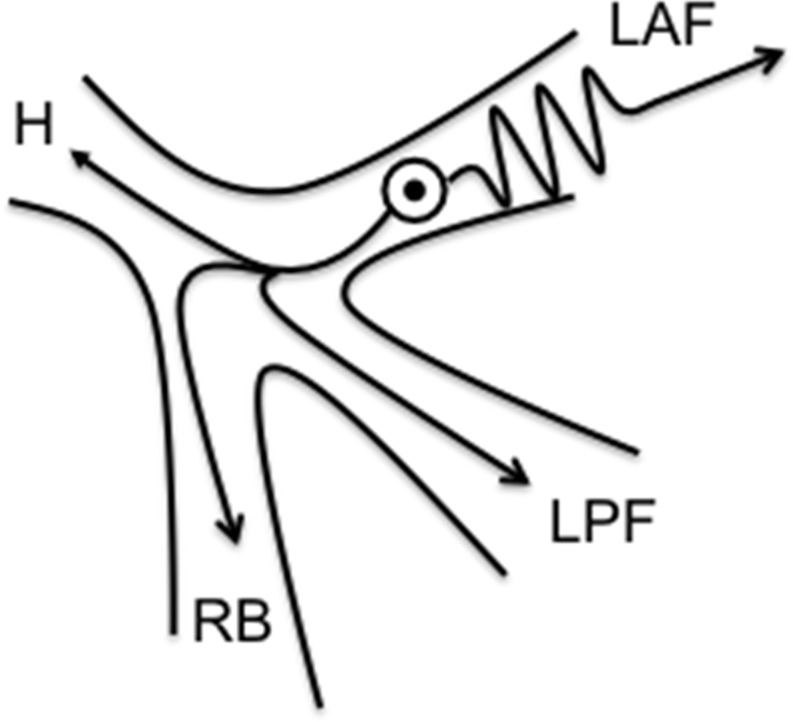
Mechanism of QRS complex by a fascicular ectopic depolarization. The speed of conduction of the impulse is delayed anterogradely over a diseased LAF. A normal speed of conduction occurs through the LPF and RB over a longer distance towards the ventricular myocardium. When both conduction times are equal, the result is a nearly synchronous activation of right and left ventricle. (*H* common His bundle, *LAF* left anterior fascicle, *LPF* left posterior fascicle, *RB* right bundle)

The premature depolarisation following the second QRS complex occurs late in the cardiac cycle (CL = 1000 ms) with a QRS duration of 120 ms followed by a complete compensatory pause (Fig. 1 in the Question article, doi: 10.1007/s12471-015-0655-z). The QRS complex is not preceded by a P wave, excluding an atrial ectopic origin. The amplitude of the R wave is large in leads V2–V4, suggesting an anterior axis deviation of the QRS complex in the precordial leads, which makes an AV nodal origin unlikely in the presence of a left anterior fascicular block during sinus rhythm. Since the QRS width is borderline at around 120 ms, a premature ventricular depolarisation cannot be excluded as origin of the ectopic focus. But what is the most likely explanation for this depolarisation?

Figure 1 shows the proposed mechanism of activation from a spot in the specific His-Purkinje system where the time of slow anterograde conduction over a diseased left anterior fascicle is equal to the normal conduction time of the impulse, travelling over a longer distance through the posterior fascicle and right bundle. This results in an almost synchronous activation of both right and left ventricle. Already in 1973 [1], electrophysiological investigations were performed to prove this paradoxical phenomenon of premature beats in patients with conduction disease in the left bundle branch (Fig. 1).

The second part of the question was why does the QRS complex following the first sinus beat in V1–V6 have a different QRS morphology than the other QRS complexes during sinus rhythm? Most likely this beat is a fusion beat, since the QRS complexes in leads V4–V6 are nearly identical with the ectopic focus from the diseased left anterior fascicle.
